# Bioprospecting and biotechnological applications of fungal laccase

**DOI:** 10.1007/s13205-015-0316-3

**Published:** 2016-01-06

**Authors:** Pooja Upadhyay, Rahul Shrivastava, Pavan Kumar Agrawal

**Affiliations:** 1 Department of Biotechnology, G. B. Pant Engineering College, Ghurdauri, Pauri, Uttarakhand India; 2 Department of Biotechnology and Bioinformatics, Jaypee University of Information Technology, Solan, HP India

**Keywords:** Laccase, Mechanism of laccase action, Laccase mediator system, Endophytic fungi, Biotechnological application

## Abstract

Laccase belongs to a small group of enzymes called the blue multicopper oxidases, having the potential ability of oxidation. It belongs to enzymes, which have innate properties of reactive radical production, but its utilization in many fields has been ignored because of its unavailability in the commercial field. There are diverse sources of laccase producing organisms like bacteria, fungi and plants. In fungi, laccase is present in Ascomycetes, Deuteromycetes, Basidiomycetes and is particularly abundant in many white-rot fungi that degrade lignin. Laccases can degrade both phenolic and non-phenolic compounds. They also have the ability to detoxify a range of environmental pollutants. Due to their property to detoxify a range of pollutants, they have been used for several purposes in many industries including paper, pulp, textile and petrochemical industries. Some other application of laccase includes in food processing industry, medical and health care. Recently, laccase has found applications in other fields such as in the design of biosensors and nanotechnology. The present review provides an overview of biological functions of laccase, its mechanism of action, laccase mediator system, and various biotechnological applications of laccase obtained from endophytic fungi.

## Introduction

Laccase is one of the few enzymes that have been the subject of study since the end of the last century. In 1883, laccase was first described by Yoshida when he extracted it from the exudates of the Japanese lacquer tree, *Rhus vernicifera* (Yoshida 1883). Their characteristic as a metal containing oxidase was discovered by Bertrand (1985). In 1896, both Bertrand and Laborde observed the presence of laccase in fungi for the first time (Desai and Nityanand 2011). Laccase has received lot of attention from researchers due to its ability to degrade a variety of recalcitrant pollutants. Compounds which are structurally similar to lignin can be oxidized (Thurston 1994) by fungal laccase (benzene diol: oxygen oxidoreductase, EC 1.10.3.2) along with ferroxidases (EC 1.16.3.1) and ascorbate oxidase (EC 1.10.3.3) from the family of extranuclear multicopper oxidases (MCOs), which in turn belong to the highly diverse group of blue copper proteins. MCOs typically contain two or four copper atoms per protein molecule and they catalyze oxidation reactions. In these reactions, electrons are removed from the reducing substrate molecules and transferred to oxygen in order to form water without the step of hydrogen peroxide formation (Ducros et al. 1998). Laccases have a wide substrate range, which can serve industrial purposes. The simple requirements of laccase catalysis (presence of substrate and O_2_), as well as its apparent stability and lack of inhibition (as has been observed with H_2_O_2_ for peroxidase), make this enzyme both suitable and attractive for industrial applications. In addition, laccase can oxidize a wide range of organic and inorganic substrates, including mono, di, polyphenols, aminophenols, methoxyphenols as well as metal complexes which are the major reason for their attractiveness for dozens of biotechnological applications.

Fungi can survive under marginal living conditions as they produce unusual enzymes capable of performing chemically difficult reactions (Viswanath et al. 2008). Interest in laccases has increased recently because of their potential use in the detoxification of pollutants and in bioremediation of phenolic compounds (Singh et al. 2011). These fungal enzymes can convert jet fuel (Viswanath et al. 2014), paint, plastic and wood among other materials into nutrients. Some enzymes have already been harnessed in pulp and paper processing and in the synthesis of fine chemicals (Viswanath et al. 2008).

### Mechanism of laccase action

**Fig. 1:**
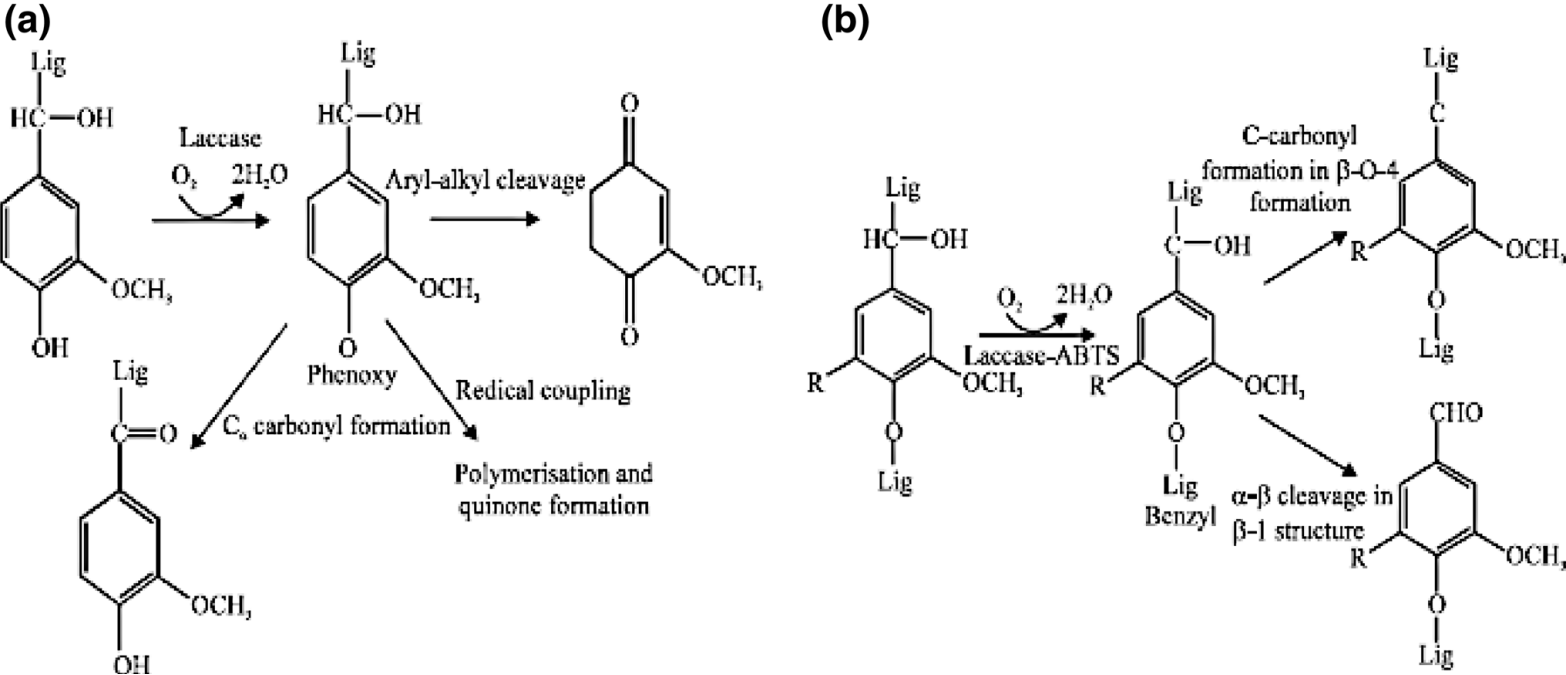
**a** Oxidation of phenolic subunits of lignin by laccase and **b** oxidation of nonphenolic lignin model compounds by a laccase mediator system (both adapted from Archibald et al. 1997)

Laccase only attacks the phenolic subunits of lignin which leads to aryl–alkyl cleavage, Cα oxidation and Cα–Cβ cleavage (Fig. 1a). Laccase catalysis comprises of the following steps:

1. Reduction of the type 1 copper by reducing substrate.
2. Internal electron transfer from the type 1 to the type 2 and type 3 copper.
3. Reduction of oxygen to water at the types 2 and 3 copper site.

Laccase plays important role in lignin biodegradation but earlier its application was limited to phenolic compounds, because of low oxidation potential of such enzymes (Madhavi and Lele 2009). In the presence of mediator compound such enzymes show higher oxidation capability resulting in numerous biotechnological applications involving oxidation of non-phenolic lignin compounds (Khambhaty et al. 2015) (Fig. 1b).

The laccase mediator system (LMS) was first described by Bourbonnais and Paice (1990) with the use of ABTS [2,2′-azino-bis (3-ethylbenzothiazoline-6-sulphonic acid)] as the first mediator. It was originally developed to solve the problems in bio-bleaching of wood pulps. The delignification of kraft pulp by laccase can be supported by a number of low molecular mass dyes or aromatic hydrogen donors (Bourbonnais et al. 1995). ABTS was the first mediator found to be effective in the delignification of kraft pulp and lignin transformation by laccase.

The reaction mechanism mediated by ABTS proceeds as follows.

Laccase is activated by oxygen which then oxidizes the mediator. The mediator then diffuses into pulp and oxidized lignin, which disrupts it into smaller fragments, and hence they are easily removed from the pulp with the help of alkaline extraction. The application of the LMS on hardwood kraft pulp results in demethylation, depolymerization of kraft lignin and reduction of kappa number (Archibald et al. 1997; Reid and Paice 1994).

Some other applications of LMS involve oxidation of aromatic methyl groups, benzyl alcohols (Johannes et al. 1996), bleaching of textile dyes (Rodriguez et al. 2004) and polycyclic aromatic hydrocarbons (Johannes et al. 1996; Majcherczyk et al. 1998; Johannes and Majcherczyk 2000).

### Biotechnological application and biological function of laccases

Laccase can be found in plants (Dittmer et al. 2004; Shraddha et al. 2011), bacteria (Claus 2003; Abdel-Hamid et al. 2013) and insects (Kramer et al. 2001), but its major source is fungi. The biological function of laccases in fungi is still unclear, but several biological functions such as spore resistance and pigmentation (Aramayo and Timberlake 1993; Williamson et al. 1998), lignification of plant cell walls (Malley et al. 1993), humus turnover, lignin biodegradation and detoxification processes (Baldrian 2006), virulence factors, and copper and iron homeostasis (Stoj and Kosman 2003) have been proposed with a good scientific basis. Physiological function of laccase in plants is the polymerization of monolignols into dimers and trimers during lignification process (Bertrand et al. 2002). They are involved in cell wall formation in plants together with peroxidases. High levels of laccase like multi-copper oxidase (LMCO) expression in plant vascular tissues may reflect the need for high-efficiency iron uptake pumps in tissues that undergo lignifications in *Liriodendron tulipifera* (Hoopes and Dean 2004). The presence of laccases in resin ducts of anarcardiaceae suggests a rather obvious function, i.e. invasion by bacteria, fungi and defence against herbivores (Mayer and Staples 2002).

However, most biotechnologically useful laccases (i.e. those with high redox potentials) are of fungal origin (Thurston 1994). Over 60 fungal strains belonging to Ascomycetes, Basidiomycetes and Deuteromycetes show laccase activity. Among the latter group, the lignin-degrading white-rot fungi are the highest producers of laccase (Shraddha et al. 2011) but litter-decomposing and ectomycorrhizal fungi also secret laccases. In the presence of small redox mediators, laccase offers a wide repertoire of oxidations including non-phenolic substrates. Hence, fungal laccases are observed as ideal green catalysts of great biotechnological impact due to their few requirements (they exhibit water as the only by-product and require only air) and their broad substrate specificity, including direct bioelectrocatalysis (Kunamneni et al. 2008).

**Fig. 2:**
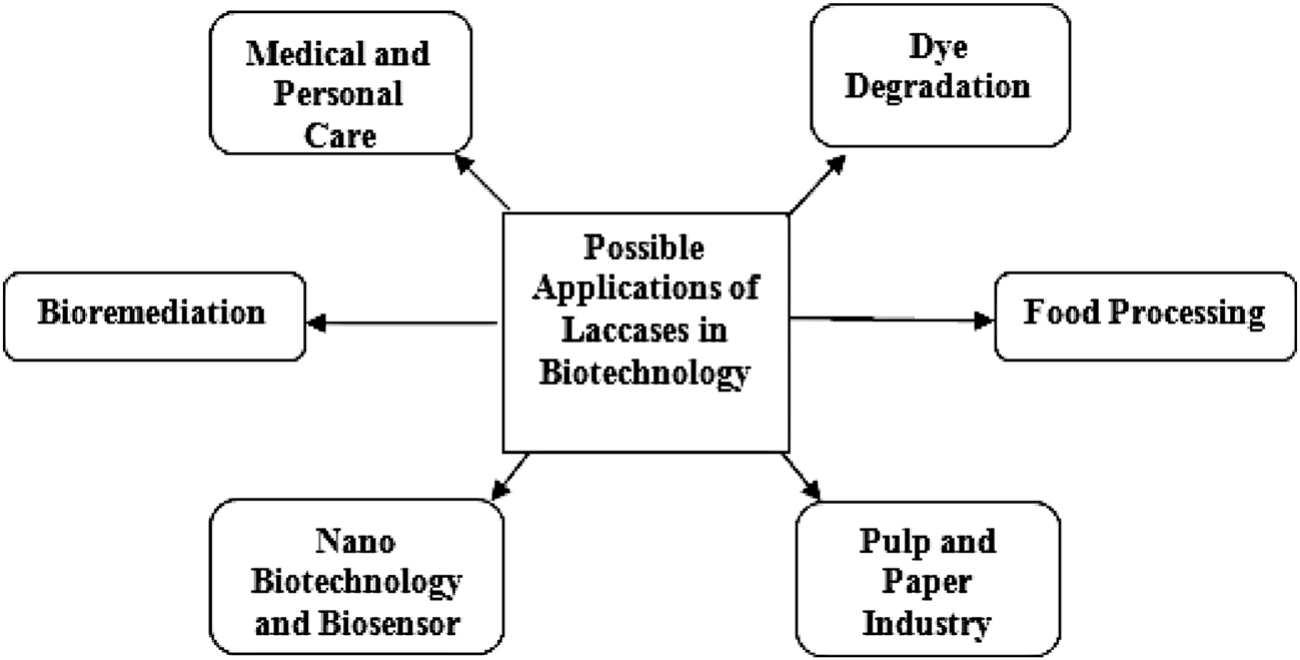
Application of laccase in biotechnology

The biotechnological applicability of laccase may therefore be extended by the use of laccase-mediator system (LMS). Thus, laccase and LMS find potential application in delignification (Virk et al. 2012), and biobleaching of pulp (Ibarra et al. 2006; Weirick et al. 2014); enzymatic modification of dye-bleaching and fibres in the textile and dye industries (Kunamneni et al. 2008); enzymatic removal of phenolic compounds in beverages—wine and beer stabilization, fruit juice processing (Minussi et al. 2002); enzymatic cross linking of lignin-based materials to produce medium density fibreboards (Widsten et al. 2004); bioremediation and detoxification of aromatic pollutants (Alcalde et al. 2006; Khambhaty et al. 2015); detoxification of lignocellulose hydrolysates for ethanol production by yeast (Larsson et al. 1999); treatment of industrial wastewater (Berrio et al. 2007; Viswanath et al. 2014) and construction of biofuel cells and biosensors (Ghindilis 2000; Shraddha et al. 2011). Due to its catalytical properties, laccase has gained considerable interest for potential biotechnological applicability (Bourbonnais et al. 1995; Abdel-Hamid et al. 2013). Various biotechnological applications of laccase cited in this review paper are represented in Fig. 2 (Table 1).

**Table 1:** Biotechnological applications of fungal laccase

S. no.	Source of laccase	Applications	References
1.	*Aspergillus flavus*	Decolorization of Malachite green dye	Ali et al. (2009)
2.	*Coriolus versicolor*	Degradation of textile dyes	Sanghi et al. (2009)
3.	*Streptomyces cyaneus*	Decolorize and detoxify azo dyes	Moya et al. (2010)
4.	*Phanerochaete chrysosporium*	Decolorize commercially used reactive textile dyes; reactive orange 96, reactive violet 5, reactive black 5 and reactive blue 38	Heinfling et al. (1997)
5.	*Schizophyllum commune*	Decolorizing wastewater released from a bagasse-pulping plant	Belsare and Prasad (1988)
6.	*Pycnoporus cinnabarinus*	Decolorizing of pigment plant effluent	Schliephake et al. (1993)
7.	*Coriolopsis gallica*	Oxidized recalcitrant polycyclic heterocycles compounds carbozole, *N*-ethylcarbozole, fluorine, and dibenzothiophene present in coal tar and crude oilin presence of 1-hydroxybenzotriazole and 2,2′-azinobis (3-ethylbenzthiazoline)-6-sulfonic acid as free radical mediators	Dec and Bollag (2000)
8.	*Pichia pastoris*	Engineered to improve the efficiency of particular bioremediation processes	Dhawan and Kuhad (2002)
9.	*Pleurotus ostreatus*	Degradation of polycyclic aromatic hydrocarbons in the presence of a synthetic mediator	Pozdnyakova et al. (2006)
10.	*Pleurotus eryngii*	Lignin and organopollutant degradation, as well as to improve the bioremediation potential	Gomez-Toribio et al. (2009)
11.	*Trametes pubescens*	Bioremediation of a mixture of pentachlorophenol (PCP), 2-chlorophenol (2-CP), 2,4-dichlorophenol (2,4-DCP) and 2,4,6-trichlorophenol (2,4,6-TCP)	Gaitan et al. (2011)
12.	*Marasmius quercophilus*	Biotransformation of alkylphenols	Farnet et al. (2000)
13.	*Trametes versicolor*	Wine stabilization	Minussi et al. (2007)
14.	*Myceliophthora thermophila*	Dough conditioner	Renzetti et al. (2010)
15.	*Coriolopsis gallica*	Beer factory waste water treatment	Madhavi and Lele (2009)
16.	*Pycnoporus cinnabarinus*	Processing aid for the food industry	Li et al. (1999)
17.	*Botrytis cinerea*	Processing aid for the food industry	Li et al. (1999)
18.	*Phaenerochaaete chrysosporium*	Dechlorination and decolorization of pulp and paper effluent	Eaton et al. (1980)
19.	*Coriolus versicolor*	Increased brightness of hardwood kraft pulp	Livernoche et al. (1983)
20.	*Trametes versicolor*	Paper biosensor for the detection of phenolic compounds	Oktem et al. (2012)

Fungal source of laccases and their major applications within different industrial and biotechnological prospects have been discussed as under.

#### Lignin degradation in pulp and paper industry

In a piece of wood, the wood fibres are glued together by lignin. These fibres can be separated either by degradation and removal of lignin (chemical pulping) or by physically tearing the fibre apart (mechanical pulping). Separation of wood fibres from each other and then processing them into sheets leads to the formation of paper from wood (Madhavi and Lele 2009). Pulp bleaching is currently achieved by treating pulps with chlorine-based chemicals. This results in the formation of chlorinated aliphatic and aromatic compounds that could be carcinogenic, mutagenic and toxic (Taspinar and Kolankaya 1998). In the recent years, intensive studies have been performed to develop enzymatic, environmentally benign, bleaching technologies. Biobleaching of pulp has been shown by laccase mediated systems, but the feasibility of its use is hindered by the lack of an inexpensive mediator (Taspinar and Kolankaya 1998).

Laccases can depolymerize lignin and delignify wood pulps (Virk et al. 2012) due to its property of removing potentially toxic phenols arising during degradation of lignin. Firstly laccase acts on small phenolic lignin fragments that react with the lignin polymer, and which then results into its degradation. Moreover, pretreatment of wood chips with ligninolytic fungi increases the pulp strength while energy requirement for mechanical pulping is decreased. Some other uses of laccases for the pulp and paper industry include reduction of the kappa number of pulp (Abdullah et al. 2000; Singhal et al. 2005) and an improvement in the paper making properties of pulp (Kuznetsov et al. 2001). Laccase producing *C. albidus* was effective in reducing the lignin content of eucalyptus wood and can be used for biopulping in the pulp and paper industry (Singhal et al. 2005). Pretreatment of hardwood with *Phlebia tremellosa* produced an 80 % increase in the tensile strength. Energy requirement was decreased by 47 % by incubating aspen chips for 4 weeks with *Phlebia brevispora*. Fungal laccases can be used for the treatment of effluents from pulp mills or from other industries containing chlorolignins or phenolic compounds. Laccases render phenolic compounds less toxic via degradation or polymerization reactions and/or cross-coupling of pollutant phenol with naturally occurring phenols. Laccase base biobleaching process offers an environmentally benign way to improve pulp and paper production.

#### Dye degradation

The textile industry accounts for two-thirds of the total dyestuff market and consumes large volumes of water and chemicals for wet processing of textiles (Banat et al. 1996). Textile dyeing effluents containing recalcitrant dyes pollute water due to their color and by the formation of toxic or carcinogenic intermediates such as aromatic amines from azo dyes. The chemical reagents used are very diverse in chemical composition, ranging from inorganic compounds to polymers and organic products (Zollinger 2002). Poots et al. (1976) reported that commercially there are about 1,00,000 available dyes with over 7 × 10^5^ tonnes of dyestuff produced annually. Dyes are usually resistant to fading on exposure to light, water and also to various chemicals due to their complex chemical structure, and most of them are difficult to decolourise due to their synthetic origin (Lin et al. 2010a, b).

Synthetic dyes are extensively used in various industries. The problems related with the release of coloured effluents from different industries such as food, paper, plastics textiles and cosmetics are great concerns for various scientists (Mahmoodi et al. 2009).

Since conventional treatment systems based on chemical or physical methods are quite expensive and consume high amounts of chemicals and energy, alternative technologies for this purpose have recently been studied. Laccase in this field has received particular attention because of its ability to catalyze the oxidation of a wide spectrum of pollutants. A number of anaerobic and aerobic processes have been developed at laboratory scale to treat dyestuff by various researchers. Fungal cultures belonging to white rot fungi have been extensively studied to develop bioprocesses for the mineralization of azo dyes. White-rot fungi are a class of microorganisms that produce efficient enzymes capable of decomposing dyes under aerobic conditions. They produce various oxidoreductases that degrade lignin and related aromatic compounds (Ali 2010; Khan et al. 2013). White rot fungi do not require preconditioning to particular pollutants, because enzyme secretion depends on nutrient limitation, rather than presence of pollutant. Sanghi et al. (2009) studied the potential of white-rot fungal strain *Coriolus versicolor* to decolorize five structurally different dyes in sequential batch reactors under optimized conditions and the decolourization potential of the fungus was mainly dependent on the nutrients and composition of the medium. Although stable operation of continuous fungal bioreactors for the treatment of synthetic dye solutions has been achieved, application of white rot fungi for the removal of dyes from textile wastewater faces many problems such as large volumes produced, the nature of synthetic dyes, and control of biomass (Elisangel et al. 2009). The ability of a laccase produced by *Streptomyces cyaneus* to decolourise and detoxify azo dyes was analysed. 90 % colour loss was attained only in the presence of acetosyringone acting as a redox mediator for laccase (Moya et al. 2010). Laccases potential of acting on chromophore compounds such as azo, anthraquinone, heterocyclic, indigoid dyes, polymeric dyes, remazol brilliant blue R, triarylmethane and triphenylmethane leads to the suggestion that they can be applied in industrial decolourization processes (Abdullah et al. 2000). Due to their high specificity, enzymes only attack the dye molecules while valuable dyeing additives or fibers are kept intact and can be reused. Both microorganisms and isolated enzymes have a high potential for the treatment of process effluents in the textile industry to allow their reuse. Currently, there many researchers have optimized dye decolourization using laccase (Fazli et al. 2010; Sharma et al. 2009).

Most existing processes to treat dye wastewater are ineffective and not economical. Hence, fungal decolurization of dye by using laccase can provide an attractive solution due to their potential in degrading dyes of diverse chemical structure including synthetic dyes. This enzyme decolorizes some azo dyes without direct cleavage of the azo bonds through a highly non-specific free radical mechanism, thereby avoiding the formation of toxic aromatic amines (Kalme et al. 2009). Use of fungal biomass to remove colour in dye of industrial effluent is still in research stage and the described system has to be developed with a cheaper biocatalyst that would be cost effective. Thus, laccase based dyes treatment may provide a reasonable basis for the development of biotechnological processes for continuous color and aromatic compounds removal from various industrial effluents at large scale in the industry.

#### Application of laccase in bioremediation

One of the major problems, besetting the world today, is the contamination of air, soil and water, by harmful chemicals which may have catastrophic impact on human health and environment. The pollution of the environment has become a serious issue with industrialization and immense use of pesticides in agriculture. Around 80 billion pounds of perilous organopollutants are manufactured annually in the US alone and only 10 % of these are disposed of safely (Reddy and Mathew 2001). Stringent regulations have been imposed on industries to treat their waste effluents prior to their final discharge in the environment.

Several remediation techniques have been reported during the past two decades, but very few of them have been accepted by some industries. Certain perilous compounds, such as pentachlorophenols (PCP), polychlorinated biphenyls (PCB), polycyclic aromatic hydrocarbons (PAH), 1,1,1-trichloro-2,2-bis(4-chlorophenyl) ethane (DDT), benzene, toluene, ethylbenzene, and xylene (BTEX) as well as trinitrotoluene (TNT), are persistent in the environment and known to have carcinogenic and/or mutagenic effects. The fungal ability to transmute a vast variety of hazardous chemicals has aroused interest in using them in bioremediation (Bollag et al. 2003). Biological processes have attracted as a viable alternative to the known chemico-physical methods due to their cost, effectiveness and environmental benignity. These processes have potential to mineralize dyes to harmless inorganic compounds like CO_2_, H_2_O and the formation of a lesser quantity of sludge (Mohan et al. 2002). Many researchers have demonstrated partial or complete biodegradation of xenobiotics by pure or mixed cultures of endophytic fungi.

In the early 1980s, researchers had developed the idea of using oxidoreductases for the remediation of water contaminated by aromatic pollutants. Enzymatic remediation has an advantage because these enzymes can act on a broad range of substrates, like phenols, chlorophenols, methylated phenols, bisphenols, anilines, benzidines and other heterocyclic aromatic compounds under dilute conditions and are less sensitive to operational upsets than the microbial populations (Husain and Husain 2008). Enzymatic treatment is currently considered as an alternative method for the removal of toxic xenobiotics from the environment (Marbach et al. 1985). Fungal laccases are used in the decolourization and detoxifications of effluents released from industries, and also help in the treatment of wastewater (Chandra and Chowdhary 2015). They act on both phenolic and nonphenolic lignin-related compounds as well as highly recalcitrant environmental pollutants, and can be effectively used in textile industries, pulp and paper industries, bioremediation and xenobiotic degradation (Viswanath et al. 2014). Potential environmental application for laccases is the bioremediation of contaminated soils as laccases and immobilized laccases are able to oxidize toxic organic pollutants, such as chlorophenols, polycyclic aromatic hydrocarbons, lignin related structures, organophosphorus compounds, phenols and azo dyes (Leonowicz and Trojanowski 1975; Saratale et al. 2011; Khan et al. 2013).

Laccaseare blue multicopper oxidases, which oxidize or polymerize phenolic compounds to less toxic compounds (Viswanath et al. 2014). Laccase substrates comprise of phenols, dyes, polycyclic aromatic hydrocarbons, endocrine disrupters and pesticides, out of which some can be oxidized by extracellular fungal laccase (Majeau et al. 2010). Induction of OH^−·^ production through quinone redox cycling enabled *Pleurotus eryngii* to oxidize the dye reactive black 5 and phenol, obtaining a high linkup between the rates of pollutant oxidation and OH^−^ production (Gomez-Toribio et al. 2009).

The biodegradation of a mixture of pentachlorophenol (PCP), 2-chlorophenol (2-CP), 2,4-dichlorophenol (2,4-DCP) and 2,4,6-trichlorophenol (2,4,6-TCP) using the laccase produced by the white-rot fungal strain *Trametes pubescens* was evaluated by Gaitan et al. (2011). The laccase potential from the white-rot fungus *Marasmius quercophilus* to transform certain alkylphenols (*p*-nonylphenol, *p*-octylphenol and *p*–*t*-octylphenol) was studied by Farnet et al. (2000). The white-rot fungus *Trametes vesicolor* degraded naproxen in a liquid medium to non-detectable levels after 6 h. In vitro degradation experiments with purified laccase and purified laccase plus mediator 1-hydroxybenzotriazole (HBT) showed slight and almost complete naproxen degradation (Marco-Urrea et al. 2010). The detoxification of water soluble fraction named as “alpeorujo” a by-product obtained in olive oil extraction industry by laccase was reported by Saparrat et al. (2010). Zhao et al. (2010) showed that Laccase was responsible for biodegradation of dichlorodiphenyltrichloroethane (DDT) in soil (Zhao et al. 2010; Chao et al. 2008). Bhattacharya et al. (2009) reported laccase mediated biodegradation of 2,4-dichlorophenol and laccase treatment impairs bisphenol-A induced cancer cell proliferation (Bolli et al. 2008). Pozdnyakova et al. (2006) showed the degradation of polycyclic aromatic hydrocarbons (PAHs) like anthracene, fluoranthene, fluorene, perylene, phenanthrene and pyrene using laccase produced by *Pleurotus ostreatus* in the presence of a synthetic mediator.

Laccase is usually studied because of its ability to degrade phenolic compounds in industrial waste water (Gianfreda et al. 1999). Major classes of pollutants are aromatic amines and phenols which are highly regulated in many countries (Karam and Nicell 1997). The presence of such compounds in drinking water, irrigation water or in cultivated land is perilous for health. Laccase immobilization on polyethersulphone showed chemical and physical properties potentially useful for decreasing phenol concentration in a model wastewater treatment (Lante et al. 2000). Crecchio et al. (1995) reported that laccase removes naturally occurring and xenobiotic aromatic compounds from aqueous suspensions when it is immobilized on organogel supports. Yague et al. (2000) reported that this high tannin containing waste water was degraded by *Coriolopsis gallica*, a laccase producing white rot fungus.

#### Applications of laccase in food processing

Laccases possess great potential to be used as processing aids for the food industry, as food additives in food processing (Osma et al. 2010). Being energy-saving and biodegradable, laccase-based biocatalysts fit well with the development of highly efficient, sustainable, and eco-friendly food industries. They are increasingly being used in food industry for production of cost-effective and healthy foods (Brijwani et al. 2010). Laccases catalyzed-oxidation depends on the redox potential of the type-I copper, typically ranging between 500 and 800 mV. However, in presence of mediators, laccases are able to oxidize a wider range of substrates. Owing to the higher redox potential (+800 mV) of fungal laccases compared to plants or bacterial laccases they are implicated in several biotechnological applications related to food processing. Laccases have the potential to make food processing more economical and environmental friendly. To proficiently realize this potential it would require more efficient laccase production systems and better understanding of their mode of action. For instance, redox potentials of laccases from common laccase producing fungi are reported as 450 mV (*Myceliophthora thermophila*), 750 mV (*Pycnoporus cinnabarinus*), 780 mV (*Botrytis cinerea*) and 790 mV (*Trametes villosa*) (Li et al. 1999). With the use of mediators it is possible to extend the role of laccase to nonphenolic substrates. The versatility of laccase in its action and its wide occurrence in several species of fungi contribute to the easy applicability in biotechnological processes.

It is well known that browning is one of the major faults in beverages. The susceptibility of browning during storage is increased by laccase treatment (Gokmen et al. 1998). Colour stability is found to be greatly increased in fruit juices after treatment with laccase and active filtration, although turbidity is present. After treatment with laccase, the phenolic content of juices has been found to be greatly reduced along with an increase in colour stability (Ribeiro et al. 2010).

In the bread making process, it is a practice to add dough improvement additives to the bread dough, which results in improved flavor, texture, volume, and freshness of the bread/dough. Laccase exerts an oxidizing effect on the constituents of dough which then improves the strength of gluten structures in baked products. Use of laccase in dough results in an improved crumb structure, an increased volume, stability, strength, softness of the baked product and reduced stickiness. Laccase reduces the dough extensibility in both flour and gluten dough, as well as increases the resistance of dough to its maximum (Selinheimo et al. 2006). The addition of laccase to oat flour leads to an increased loaf specific volume and addition of proteolytic enzymes to it, simultaneously crumb hardness and chewiness is reduced which results to significant improvement to texture quality of oat bread.

Jurado et al. (2009) reported that the phenolic compounds are reduced by the induction of laccase, which act as inhibitor of fermentation and increases the production of ethanol from steam exploded wheat straw. Laccase expressing yeast strains showed a definite advantage for producing ethanol from lignocelluloses because it enabled faster growth and ethanol formation as it had the ability to convert coniferyl aldehyde at a very fast rate (Larsson et al. 1999).

Laccase is commonly used to stabilize fruit juices. Naturally occurring phenolics and their oxidation products are found to be present in many fruit juices, which add color and taste to it. The natural co-oxidation reactions and polymerization of phenols and polyphenols result in undesirable changes in aroma and color. The color change is observed as enzymatic darkening, which increases due to a higher concentration of polyphenols naturally present in fruit juices (Ribeiro et al. 2010). Research by Giovanelli and Ravasini (1993) utilized laccase in combination with filtration in the stabilization of apple juice. Laccase treatment causes removal of phenols with higher efficiency compared to other methods, like activated coals (Brijwani et al. 2010). The removal of substrate–enzyme complex is done by membrane filtration. Color stability was found to be greatly increased after treatment with laccase and active filtration, while turbidity was present. Ribeiro et al. (2010) reported that the phenolic content of juices was found to be greatly reduced after treatment with laccase along with an increase in color stability. Compared to conventional treatments, such as addition of ascorbic acid and sulphites laccase treatment has also been found to be more effective for color and flavor stability (Minussi et al. 2002).

The use of laccase enzymes allows for the improvement of functionality along with sensory properties. The successful application of laccases in food processing would require production of high amounts at low-cost. Many production strategies can be adopted along with media and process optimization to achieve better process economics. Media optimization and use of appropriate inducers could bring additional benefits of higher production with expenditure of minimum resources. Both submerged and solid state cultivation techniques have been embraced by the researchers for laccase production. Submerged fermentation, though, leads the SSF for industrial production of laccase. Future efforts in improving the SSF bioreactor designs can make SSF more potent and competitive. Laccase have important role to play in green food-based processing including beverage (fruit juice, wine and beer) stabilization, role in improvement of overall food quality, uses in baking industry and bioremediation. Ligninolytic fungi and their enzymes exhibited several biophysics and biochemical characteristics that have been applied in the delignification and detoxification of biofuel feedstocks. Ligninolytic processes (enzymatic and fungal) produce ethanol yield, glucose conversion, and delignification and detoxification percentages similar or superior than conventional detoxification and delignification methodologies (Plácido and Capareda. 2015). It is clear from the survey of literature that focus has been on a few laccase producing endophytic fungi, but there are still large numbers of fungi not yet exploited for laccase production. Search and screening of unexplored fungal species along with known and reference cultures is required to select potential cultures producing laccase in larger amounts. Further understanding of kinetic parameters of laccases will be useful for practical applications of the enzyme.

#### Medical and personal care application

Many products generated by laccases are antimicrobial, detoxifying, or active personal-care agents. Due to their specificity and bio-based nature, potential applications of laccases in the field are attracting active research efforts. Laccase can be used in the synthesis of complex medical compounds as anesthetics, anti-inflammatory, antibiotics, sedatives, etc., including triazolo(benzo)cycloalkyl thiadiazines, vinblastine, mitomycin, penicillin X dimer, cephalosporins, and dimerized vindoline.

Poison ivy dermatitis is caused mainly by urushiol, which is a catechol derivative toxin. Laccase oxidizes, detoxifies and polymerizes urushiol (Cheong et al. 2010), which reduces the effect of poison ivy dermatitis (oxidized urushiol is non-toxic). Laccase can oxidize iodide to produce iodine which is widely used as a disinfectant. It has several advantages over direct iodine application. In terms of handling, storage and transport, Iodide salt is more stable and much safer as compared to iodine. Release of iodine from a laccase iodide system could be easily controlled. The system may be used in several medical, industrial, domestic, and other personal care applications. A novel application field for laccases is in cosmetics. For example, laccase-based hair dyes could be less irritant and easier to handle than current hair dyes. Couto and Herrera (2006) reported that laccase based system may overcome drawbacks of chemical dyes by replacing hydrogen peroxide as the oxidizing agent in the dyeing formula. More recently, cosmetic and dermatological preparations containing proteins for skin lightening have also been developed. Laccases may find use as deodorants for personal-hygiene products, including toothpaste, mouthwash, detergent, soap, and diapers. Protein engineered laccase may also be used to reduce allergenicity.

The synthesis of immunomodulatory prostaglandins by the use of laccase was reported by Erb-Downward et al. (2008). Proliferation of murine leukemia cell line L1210 and human hepatoma cell line HepG2 was inhibited by laccase isolated from *Pleurotus cornucopiae*, and the activity of HIV-1 reverse transcriptase with an IC50 of 22 μM was reduced. Wong et al. (2010) reported that there was neither mitogenic activity towards mouse splenocytes nor hemagglutinating/hemolytic activity toward rabbit erythrocytes. Inhibition of hepatitis C virus entry into peripheral blood cells and hepatoma cells by laccase purified from oyster mushroom (El-Fakharany et al. 2010). From the fresh fruiting bodies of the edible white common *Agrocybe cylindracea* mushroom (Hu et al. 2011), a laccase, with HIV-1 reverse transcriptase inhibitor activity and antiproliferative activity against HepG2 and MCF7 cells was isolated.

#### Nanobiotechnology and biosensor

Laccase (EC 1.10.3.2, *p*-benzenediol: oxygen oxidoreductase) catalyzes the oxidation of various aromatic compounds, particularly phenols, which are organic pollutants, present in wastewater. With this specific function, this enzyme has a great impact on the development of biosensors for both environmentally important pollutants and clinically relevant metabolites. Laccases are able to catalyse electron transfer reactions without additional cofactors. In Biosensor technology the use of laccase is mainly attributed to its broad substrate range allowing for the detection of a broad range of phenolics, oxygen or azides; this does however disallow the detection of specific constituents (Fogel and Limson 2013). Biosensors that utilize laccase include an electrode that may be used for the detection of phenols, such as catechols in tea (Palmore and Kim 1999) and real water samples (Li et al. 2014), polyphenolic compounds in wine (Lanzellotto et al. 2014), and lignins and phenols in wastewaters (Giovanelli and Ravasini 1993). Nanostructured enzyme-based biosensor on fullerene and gold nanoparticles was performed evaluating the detection of polyphenols either in buffer solution or in real wine samples (Lanzellotto et al. 2014). Lin et al. (2010a, b) reported a non-oxidative electrochemical approach to online measurements of dopamine release through laccase-catalyzed oxidation and intramolecular cyclization of dopamine. Polyphenol index in wines is determined by laccase coupled multi-walled carbon nano tubes based biosensor. This biosensor shows reliable and fast amperometric responses to gallic acid (Di Fusco et al. 2010). Ultrasensitive amperometric detection of the catecholamine neurotransmitters dopamine, epinephrine and norepinephrine is achieved by an enzyme electrode based on the co-immobilisation of an osmium redox polymer and a laccase on glassy carbon electrodes, attaining nanomolar detection limits (Ferry and Leech 2005). For the immobilization of laccase, the hybrid material of nafion/sol–gel silicate has altered the selectivity of the enzyme to various phenolic compounds such as catechol, guaiacol, *m*-cresol and *o*-cresol (Abdullah et al. 2007). Franzoi et al. (2009) reported laccase based biosensor for determination of rosmarinic acid in plant extracts.

## Conclusion

Laccases are receiving much attention from researchers around the globe because of their specific nature. They have many industrial applications because of their innate ability of oxidation of a broad range of phenolic and non-phenolic compounds. The biotechnological significance of laccase enzymes has led to a drastic increase in the demand for these enzymes in the recent time. Laccase are promising to replace the conventional chemical processes of several industries. The introduction of the laccase-mediator system provides a biological alternative to traditional chlorine bleaching processes.

One of the limitations to the large-scale application of the enzyme is the lack of capacity to produce large volumes of highly active enzyme. These problems can be solved with the use of recombinant organisms or screening for natural hypersecretory strains. Environmental factors influence the ability of fungi to produce high titres of laccase, and different strains react differently to these conditions. One should thus select a strain capable of producing high concentrations of a suitable enzyme and then optimize conditions for laccase production by the selected organism. The effective catalytic properties of enzymes have already promoted their introduction into several industrial products and processes. Recent developments in biotechnology, particularly in areas such as protein engineering and directed evolution, have provided important tools for the efficient development of new enzymes. This has resulted in the development of enzymes with improved properties for established technical applications and in the production of new enzymes tailor-made for entirely new areas of application where enzymes have not previously been used.
